# YAP1 is essential for malignant mesothelioma tumor maintenance

**DOI:** 10.1186/s12885-022-09686-y

**Published:** 2022-06-10

**Authors:** Loreley Calvet, Odette Dos-Santos, Emmanuel Spanakis, Véronique Jean-Baptiste, Jean-Christophe Le Bail, Armelle Buzy, Pascal Paul, Christophe Henry, Sandrine Valence, Colette Dib, Jack Pollard, Sukhvinder Sidhu, Jürgen Moll, Laurent Debussche, Iris Valtingojer

**Affiliations:** 1 Department of Oncology, In Vivo Pharmacology, Sanofi Research Center, Vitry-sur-Seine, France; 2 Department of Oncology, Molecular Oncology, Sanofi Research Center, Vitry-sur-Seine, France; 3 Department of Oncology, Precision Oncology, Sanofi Research Center, Vitry-sur-Seine, France; 4 Department of Translational Sciences, Sanofi Research Center, Chilly Mazarin, France; 5 Department of Oncology, Precision Oncology, Sanofi Research Center, Cambridge, USA

**Keywords:** Malignant mesothelioma, YAP1, YAP-TEAD transcription signature, Tumor maintenance

## Abstract

Malignant pleural mesothelioma, a tumor arising from the membrane covering the lungs and the inner side of the ribs, is a cancer in which genetic alterations of genes encoding proteins that act on or are part of the Hippo-YAP1 signaling pathway are frequent. Dysfunctional Hippo signaling may result in aberrant activation of the transcriptional coactivator protein YAP1, which binds to and activates transcription factors of the TEAD family. Recent studies have associated elevated YAP1 protein activity with a poor prognosis of malignant mesothelioma and its resistance to current therapies, but its role in tumor maintenance is unclear. In this study, we investigate the dependence of malignant mesothelioma on YAP1 signaling to maintain fully established tumors *in vivo*. We show that downregulation of YAP1 in a dysfunctional Hippo genetic background results in the inhibition of YAP1/TEAD-dependent gene expression, the induction of apoptosis, and the inhibition of tumor cell growth *in vitro*. The conditional downregulation of YAP1 in established tumor xenografts leads to the inhibition of YAP1-dependent gene transcription and eventually tumor regression. This effect is only seen in the YAP1-activated MSTO-211H mesothelioma xenograft model, but not in the Hippo-independent HCT116 colon cancer xenograft model. Our data demonstrate that, in the context of a Hippo pathway mutated background, YAP1 activity alone is enough to maintain the growth of established tumors *in vivo*, thus validating the concept of inhibiting the activated YAP1-TEAD complex for the treatment of malignant pleural mesothelioma patients.

**Supplementary Information:**

The online version contains supplementary material available at 10.1186/s12885-022-09686-y.

## Introduction

Malignant mesothelioma is a highly lethal cancer of serosal membranes predominantly from the pleural cavities (malignant pleural mesothelioma; MPM). Its development is almost exclusively associated with asbestos exposure, but other environmental pollutes have also been implicated. In addition, germline mutations of BRCA1-associated protein (BAP1) and of other tumor suppressor genes may interact with asbestos exposure as causal effects [1]. MPM is a rare cancer type with an estimated 10,000 to 15,000 annual cases worldwide and shows a long latency period of 20–50 years between asbestos exposure and cancer development [2]. Due to the rarity of the disease, the long latency, and the non-specific presentation of symptoms such as cough and chest pain, disease diagnosis is often delayed. Despite of a limit or ban of asbestos in most industrialized countries since the 1990s, MPM incidence and mortality worldwide have continued to increase as many countries, such as the Russian Federation, India, and China, or South American keep using asbestos [1]. Treatment modalities rely on chemotherapy-, and radiotherapy, and most recently the use of immune checkpoint inhibitor combinations. However, treatment benefit remains limited with an observed median survival of 10 to 17 months and a five-year overall survival rate of 10% [3]. The lack of efficacious treatment options provides a compelling medical need for new therapies. Recently, genetic alterations in the HIPPO-YAP1 signaling cascade have been reported in MPM.

The Hippo-YAP1 signaling pathway is evolutionarily conserved and integrates intrinsic and extrinsic signals, such as mechanical force, cell–cell contact, polarity, and stress. In mammals, its core signaling cascade consists of the serine-threonine kinases STK3/4 and LATS1/2, their associated adaptor proteins SAV1 and MOB1A, and upstream regulators such as NF2 and multiple G protein-coupled receptors [4]. YAP1 (YES-associated protein 1) is a central signaling hub for the Hippo pathway. It acts as a transcription co-factor that associates preferentially with the proteins of the transcriptional enhancer associated domain (TEAD) family genes (TEAD1, TEAD2, TEAD3, and TEAD4) to form a functional transcription activator complex. In this role, YAP1 competes with its paralog WWTR1 (transcriptional co-activator with a PDZ-binding motif, gene name; commonly known as TAZ), which is regulated in an analogous way by Hippo genes [5]. The YAP1/TAZ-TEAD complex controls the transcription of genes for cell proliferation, cell survival, cell plasticity, and cell migration. Upon Hippo activation, YAP1 and TAZ are phosphorylated and remain inactive through sequestration in the cytoplasm or degradation by the proteasomal machinery. Inactivation of the Hippo pathway prevents the phosphorylation of cytosolic YAP1 and TAZ, which then translocate to the nucleus, associate with TEAD transcription factors, and initiate transcription of genes for cell proliferation and survival [6].

In healthy adult human tissues, the Hippo pathway is generally active apart from exceptions such as tissue injury, where the pathway is temporarily switched off to allow activation of YAP1 for tissue repair and regeneration (reviewed in [7]). Genetic aberrations of Hippo genes and constitutive activation of YAP1 have been described in many different cancer types with varying relevance for cancer progression (reviewed in [8, 9]). MPM is an indication in which multiple genetic alterations either in the Hippo pathway itself or in regulators or effectors of the Hippo-YAP1 pathway have been reported, notably loss-of-function mutations in NF2 [10, 11], an upstream regulator of Hippo-YAP1, or BAP1, a regulator of LATS2, or LATS2 itself, a core component of the Hippo signaling kinase cascade [12–14]. The prevalence of these genetic alterations sums up to more than 50% of the patients of the mesothelioma cohort (MESO) of The Cancer Genome Atlas (TCGA; US National Cancer Institute, https://www.cancer.gov) and suggests a role for YAP1 activity in MPM tumor growth.

To determine the relevance of YAP1 in MPM, we explored the role of YAP1 on tumor maintenance and whether the selective inhibition of YAP1 could drive antitumor activity *in vivo*. We first established a gene transcription signature specific to YAP1/TAZ-dependent TEAD activation (YAP1-TEAD activation) and used this signature to confirm the high prevalence of YAP1-TEAD activity in MPM samples from patients. We then selected the MSTO-211H MPM cell line with a Hippo deletion and YAP1-TEAD activation and used it as a xenograft in mice to follow the effects of downregulation of YAP1 in fully established tumors. We observed, that YAP1 knockdown not only stopped tumor progression, but lead to massive tumor regression *in vivo*. According to our signature, this phenotype was accompanied by a decrease in YAP1-TEAD-dependent transcription and was specific to the Hippo-deleted and YAP1-TEAD-activated MSTO-211H model since knockdown of YAP1 in the Hippo-independent HCT116 colon cancer xenograft model had no impact on tumor growth. We further validated the relevance of Hippo targeting *in vivo*, using the recently published pharmacological TEAD palmitoylation inhibitor K-975 [15]. Our data unequivocally show that YAP1-TEAD activity inhibition is a promising therapeutic approach for patients with MPM tumors.

## Materials and methods

### Cells and culture conditions

All cell lines were maintained at Sanofi and were grown at 37 °C under 5% CO_2_^.^ MSTO-211H (#CRL-2081), NCI-H226 (#CRL-5826), NCI-H2052 (#CRL-5915), NCI-H28 (#CRL-5820), and HCT116 (#CCL-247) were purchased from the American Type Culture Collection (ATCC, Manassas, VA, USA) and cultured according to supplier’s recommendation. ZL5 (No.11120715), ZL34 (No.11120713), SPC212 (No.11120717), Mero-14 (No.09100101), Mero-48a (No.09100104), Mero-95 (No.09100108), JU77(No.10092309), LO68 (No.10092311), and ONE58 (No.10092313) were purchased from the European Collection of Authenticated Cell Cultures (ECACC, Public Health England, Salisbury, UK) and cultured according to supplier’s recommendation. A short tandem repeat assay authenticated all cell lines at the Microsynth AG (Balgach, Switzerland). PCR using the Venor®GeM kit (Biovalley, Nanterre, France) excluded mycoplasma infection.

### Generation of cell lines and constructs

We generated cell lines stably expressing sh-YAP1, sh-Null, or TEAD2-DN, by transducing the MSTO-211H and HCT116 cells with piggybac transposon vectors using the Lipofectamine 3000 reagent protocol from Thermofisher Scientific. Transduced cells were selected in media supplemented with 10 µg/ml puromycin. For the generation of piggybac constructs, DNA sequences of sh-Yap1 and sh-Null, were cloned into the pPiggyKO-TetOne-Puro_DEST plasmid. The DNA sequences of human TEAD2-DN (Ser112-Asp447; [16]) with a FLAG-tag at the N-terminus were cloned into pPiggybacA-TetOne-puro_DEST vector.

### Cell growth and apoptosis assays

Cells were seeded in medium supplemented or not with doxycycline (1 µg/ml) and were incubated for 96 h at 37 °C and 5% CO_2_. Cell growth was measured by the trypan blue dye exclusion method using Vi-CELL-XR Cell Viability Analyzer (Beckman Coulter). Caspase-3/7 activity was detected using the CellEvent Caspase-3/7 Green ReadyProbes reagent (Molecular Probes) and measured by Incucyte ZOOM live cell analysis system (Essen Bioscience) with scans every two hours for 72 h. The acquired fluorescent signal for activated caspase-3/7 was normalized with well confluency at each timepoint (= normalized apoptosis). Peak apoptosis was determined as the highest normalized caspase-3/7 activity value during the assay.

### Animals

Female CB17/lcr-Prkdc^scid^/lcrIcoCrl mice (6–8 weeks old) were bred at Charles River (Les Oncins, France), housed in Sanofi AAALAC accredited animal facilities, and were provided with irradiated food and filtered water ad libitum. All experiments were carried out following the French law and the European Directive 2010/63/EU for the protection of animals used for scientific purposes and with approval of the ethics committee #21 (project number APAFIS#5644–2,016,061,311,593,064.V1).

### *In vivo* xenograft studies

*In vivo* tumor growth of thirteen malignant mesothelioma cell lines (MSTO-211H; ZL5; NCI-H226; JU77; MERO 48A; MERO 95; H2052; ZL-34; SCP-212; MERO-14; Lo68; One58; H28) was evaluated after subcutaneous cell inoculation into the right flank of SCID mice at 3 × 10^6^ cells and 10 × 10^6^ cells. In vivo target validation studies including tumor growth and pharmacodynamic studies were conducted using the MSTO-211H-TEAD2-DN, MSTO-211H-SH-YAP1, and HCT116-SH-YAP1 cell lines inoculated subcutaneously at 3 × 10^6^ cells mixed with Matrigel. Immediately after cell implantation, mice were divided into two groups. Each group received drinking water supplemented with either doxycycline and 5% glucose or with 5% glucose only. All mice were used without any exclusion.

*In vivo* efficacy and pharmacodynamic studies of the K-975 compound were performed in SCID mice inoculated subcutaneously into the right flank with 3 × 10^6^ MSTO-211H or NCI-H226 cells. For efficacy studies mice bearing around 200 mm^3^ subcutaneous MSTO-211H tumors or 150 mm^3^ subcutaneous NCI-H226 tumors were randomly assigned to 5 groups of 8 mice/group and treated with either vehicle or K-975 compound twice daily at 30, 100, and 200 mg/kg for 18 consecutive days. Tumor perpendicular diameters were measured twice a week with a caliper, and tumor volume (V) was calculated according to the following equation: V (mm^3^) = (d^2^ (mm^2^) x D (mm))/2, where d is the smallest, and D the largest perpendicular tumor diameters. For pharmacodynamic studies, mice bearing around 300 mm^3^ subcutaneous MSTO-211H or NCI-H226 (*n* = 3 to 5 per group) were treated with either vehicle or K-975 compound at 30, 100, and 200 mg/kg single administration, tumors were sampled for analysis at 1 h, 3 h, 6 h, and 24 h post single administration. All mice were used without any exclusion.

### RNA-sequencing and data analysis

Fresh tumors were preserved in RNAlater and frozen at -200 °C. Copurification of miRNA and total RNA was performed using a miRNeasy Mini Kit (QIAGEN, 217,004). The RNA concentration and purity were evaluated with an ND-1000 spectrophotometer (NanoDrop, Thermo Fisher Scientific, Wilmington, DE, USA). Quality metrics are the RNA Integrity Number (RIN) and DV200 value (% of RNA fragments with a length ≥ 200nt). 100 ng total RNA per sample was used as input material for the library preparation. The libraries were prepared with the KAPA RNA HyperPrep Kit (Kapa Biosystems, KK8541) and KAPA Target Enrichment using the Kapa HyperCap V3.0 kit for Illumina following the manufacturer’s recommendations. Pairs-end sequencing of the libraries was performed using the NextSeq 500 platform (Illumina, Inc., San Diego, CA).

### A YAP1-TEAD activity signature

To measure YAP1-TEAD activity in tumors and to evaluate the pharmacodynamic effects of TEAD-inhibitors, we estimated the rate of Hippo-YAP1/TAZ-TEAD-dependent transcription based on a transcriptional signature. The downstream effectors of TEAD transcription were determined by differential expression analysis of various public datasets listed in Table 1. Briefly, in these experiments, the transcription factors or their negative regulators were knocked out, or in, to modulate YAP1-TEAD activity in various cell lines. We call positive or negative effectors the downstream genes of which the RNA levels correlated positively or negatively with YAP1-TEAD activity, respectively. For instance, a YAP1 knockout (KO) should decrease YAP-TEAD activity and the level of positive downstream effectors, while increasing the levels of negative effectors. Inversely, knocking out a Hippo gene, or a Hippo-activator gene, should increase YAP1 and TEAD activity, up-regulate the positive downstream effectors and down-regulate the negative effectors. From the differentially expressed genes we retained only those consistently modulated at *p* < 0.01 in the same (expected) direction in at least 2 experiments. The so derived positive and negative TEAD effectors are listed in the Supplementary Information.

**Table 1:** Public datasets used for the derivation of the YAP-TEAD transcription signature

GEO series	Tissue	Disturbed gene	Reference
GSE7700	Breast	YAP1 KO	[17]
GSE10196	Breast	YAP1 over-expression	[18]
GSE32597	Liver	YAP1 or TEAD1 KO	[19]
GSE35004	Liver	YAP1 KO	[20]
GSE41387	Gastric cancer	YAP1-S127A constitutively active	[21]
GSE41508	Colorectal	YAP1-S127D *in vivo*	[22]
GSE49384	Embryonic kidney, liver, skin	NF2, LATS2, YAP1, and/or WWTR1 KO	[23]
GSE50053	Kidney	YAP1 KO	[24]
GSE50490	Colorectal	YAP1-S127A constitutively active	[21]
GSE52439	Embryonic stem cells	WWTR1/YAP1, TEAD1-4 KO	[25]
GSE56445	Breast	WWTR1/YAP1, TEAD1-4 KO	[26]
GSE60579	Breast	YAP1-S127A constitutively active	[27]
GSE61764	Bile duct	YAP1 KO	[28]
GSE61765	Bile duct	YAP1-S127A constitutively active	[28]
GSE61989	Umbilical vein	YAP1 KO	[29]
GSE66082	Breast	WWTR1/YAP1 KO	[30]
GSE66949	Mouth/pharynx	WWTR1 and/or YAP1 KO	[31]
GSE73396	Liver	WWTR1/ YAP1-TEAD1-4 (verteporfin)	[32]

### Scoring YAP1-TEAD activity

To score YAP1-TEAD activity from the transcriptome of individual samples, we isolated the effector genes and transformed their levels into fractional ranks (IBM SPSS Statistics, Chicago, IL). We, then, computed the difference of effector ranks (deR) score as Rp – Rn, where Rp is the mean fractional rank of the positive effectors, and Rn, that of the negative ones. Essentially, this score measures the average distance between positive and negative effectors of YAP1-TEAD transcription activity.

### Real-time PCR

The complementary DNA (cDNA) was synthesized using the High-Capacity cDNA Reverse Transcription Kit from Applied Biosystems with Oligo(dt) probes from Eurogentech. For real-time PCR amplification, TaqMan gene specific primers and probes (Applied Biosystems) were used and amplified in the Applied Biosystems 7900 thermocycler according to supplier’s recommendation. RPL37 was taken as a reference gene and normalization control in all assays. The relative mRNA quantification was calculated based on the comparative cycle threshold (Ct) method.

### CYR61 and CTGF proteins characterization by WES

Resected and frozen tumors were added to cell lysis buffer (1 ml/150 mg tumor, Invitrogen, FNN0011) supplemented with protease and phosphatase inhibitor cocktail (Thermo Fisher, 78,446) and were then mechanically disrupted on a Precellys 24 homogenizer (Bertin Technologies). Samples were centrifuged at 13,000 rpm for 15 min and total protein in the supernatant was quantified using the Pierce BCA optical protein assay (Pierce & Warriner). CYR61 and CTGF proteins were detected by WES (automated Western blotting from protein sample) using primary antibodies against CYR61 (14,479, Cell Signaling Technologies) and CTGF (10,095, Cell Signaling Technologies).

### Western blotting

Cells were lysed in RIPA Lysis and Extraction Buffer (89,900, Thermo Fisher) with Halt™ Protease and Phosphatase Inhibitor Cocktail (74,446, Thermo Fisher). Tumor tissue fragments were lysed with lysis buffer (Life technology) supplemented with protease phosphatase inhibitors cocktail and homogenized using a Precellys homogenizer (Bertin Technologies). The following primary antibodies were used, GAPDH (2118, Cell Signaling Technology), YAP (14,074, Cell Signaling Technology), Monoclonal anti-FLAG (F1804, Merck), Beta-tubulin (2146S, Cell Signaling Technology).

### Statistical analysis

For *in vivo* studies, a contrast analysis using Bonferroni-Holm correction for multiplicity following a two-way analysis of variance (ANOVA) Type with factors treatment and day (repeated) was performed on tumor volume changes from baseline, to compare globally and at each day, all treated groups to the control group. A probability of less than 5% (*p* < 0.05) was considered significant. Statistical analysis was performed using EverStat6 software. For cell viability, and CYR61 and CTGF characterization, a non-parametric two-tailed Student`s t test was used for comparisons between 2 groups, and non-parametric Sidak’s or Dunnett’s multiple comparisons test were used to compare more groups, respectively. Statistical analysis was performed using GraphPad Prism software 8.0.2. *, ** and *** corresponding to a *p* value < 0.05, < 0.01 and < 0.001.

## Results

### YAP1-TEAD activation is highly prevalent in MPM patients

We started by assessing the prevalence of Hippo pathway-related genetic alterations in 87 MPM samples published in the TCGA MESO collection. Genetic alteration collectively refers to homozygous deletion (GISTIC value = -2; [33], amplification (GISTIC = 2), or non-synonymous sequence mutation of a gene. The TCGA MESO collection comprises 23 cases of biphasic type and 54 epithelioid tumors. We interrogated 85 genes (listed in the Supplementary Information) which are either reported as Hippo pathway components or Hippo-regulating genes in the Kyoto Encyclopedia of Genes and Genomes (KEGG; Kanehisa Laboratories, Kyoto, Japan) or in MetaCore™ (Clarivate, Philadelphia, PA), or are established oncogenes (KRAS, EGFR), tumor suppressors (CDKN2A, CDKN2B, RB1), or otherwise mentioned in the referenced literature. In total, 56 of the 85 interrogated genes (49/74 with a documented Hippo relation) had genetic alterations, in TCGA MESO. The total prevalence of alterations in the 85 genes was 89% in the epithelioid group and 86% in the biphasic group. At least one Hippo-related alteration was found in 84% of the tumors. The most frequently altered genes were BAP1 (38%), CDKN2A (45%), CDKN2B (41%), NF2 (34%), TP53 (17%), and LATS2 (11%). The core Hippo genes NF2 and LATS2 were altered in 55% of the biphasic and 36% of the epithelioid tumors.

Using public data from experiments where the TEAD transcription co-factors or their Hippo regulators were knocked out, or in, we generated a transcriptional signature that measures the activity of TEAD. MPM was the indication with the highest scores for this signature among all TCGA tumors, and pleural mesothelioma cell lines had the highest scores among the cell lines of the CCLE collection, on average (Fig. S1A). Pooled TCGA tumors with mutations in the Hippo regulators BAP1, LATS2, and NF2 scored significantly higher than wildtype tumors (Fig. S1B). In the TCGA MESO cohort, tumors with NF2 mutation or deletion scored significantly higher than tumors with diploid, wildtype NF2 (Fig. S1C). In fact, NF2 mutation or deletion was the best predictor of the YAP1-TEAD-activity score among all tested genetic events in all the queried genes (data not shown). The YAP1-TEAD activity score was much lower in other tumor indications, e.g., cervical cancer, where YAP1-TEAD activation was also strongly associated with HIPPO-YAP1 genomic alterations [34].

### Evaluation of MPM cell lines for *in vivo* studies

To select an *in vivo* xenograft model that would be representative of the TCGA patient dataset, 13 MPM cell lines were characterized for Hippo pathway regulator alterations, TEAD1-4 expression pattern and YAP1 activation in cellular assays, and for which we subsequently determined the *in vivo* tumor growth in mice (supplementary data Table S1). All cell lines, except NCI-H28, carried Hippo pathway regulator alterations and showed YAP1 activation according to RNA signature status. In addition, all cell lines, except NCI-H28, responded to YAP1 down-regulation using YAP1 siRNA treatment *in vitro* (data not shown). Among the 13 cell lines tested, only three cell lines formed tumors when implanted as subcutaneous xenografts in mice. Tumor formation was most optimal for the MSTO-211H model with no associated body weight loss, so this model was chosen for *in vivo* target validation experiments.

### TEAD2 dominant negative (DN) expression transiently inhibits YAP1 and tumor growth *in vivo*

**Fig. 1:**
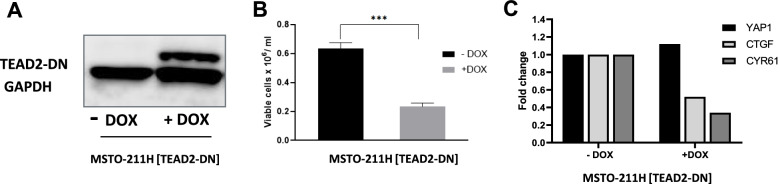
*In vitro* characterization of MSTO-211H-TEAD2-DN cells: **A** Western immunoblot analyses of TEAD2-DN in MSTO-211H-TEAD2-DN cell line 24 h post doxycycline induction. GAPDH is used as a loading control. **B** 2D cell viability of MSTO-211H-TEAD2-DN cell line 96 h post doxycycline induction. **C** RT-qPCR of YAP1 and YAP1-TEAD downstream targets CTGF and CYR61 in the MSTO-211H-TEAD2-DN cell line 72 h post doxycycline induction

The effect of YAP1 downregulation on tumor growth was first tested *in vitro* using a genetic approach based on a TEAD dominant-negative (TEAD2-DN) construct. This construct was previously reported as an efficient inhibitor of YAP1 activity [16]. It is based on a truncated version of TEAD2, which lacks the DNA binding domain but retains its ability to associate with YAP1. It thereby acts as a non-functional competitor of endogenous TEADs for binding to YAP1. We engineered this construct behind a doxycycline-inducible promoter and used it for the stable transfection of MSTO-211H cells. Upon doxycycline addition, TEAD2-DN expression resulted in 66% inhibition of tumor cell growth *in vitro*, 96 h post doxycycline induction, Fig. 1A-B, accompanied by the downregulation of target genes CYR61 and CTGF, Fig. 1C, two frequently used and direct biomarkers of YAP1-TEAD activity [35–37].

**Fig. 2:**
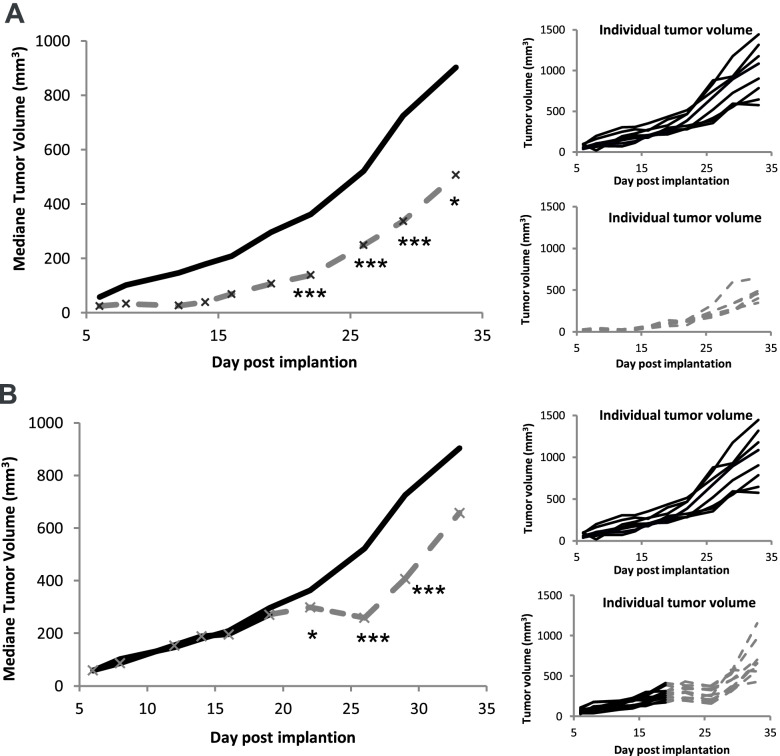
*In vivo* tumor growth of MSTO-211H-TEAD2-DN xenografts: **A** Mice were supplemented with either 5% glucose (black curve, *n* = 10) or with doxycycline (dotted grey curve, *n* = 10) in drinking water at day 0 post MSTO-211H-TEAD2-DN cell line engraftment. **B** Mice were supplemented in drinking water with 5% glucose (black curve) at day 0 post MSTO-211H-TEAD2-DN cell line engraftment, then at 19 days post engraftment, half of the mice (*n* = 10) bearing established tumors range from 171 to 414 mm^3^ were supplemented in drinking water with doxycycline (dotted grey curve). (“*” indicates adjusted *p*-value < 0.05, ** *p* < 0.01, ***p < 0.001)

To study the effects of YAP1 downregulation *in vivo*, we grafted the MSTO-211H-TEAD2-DN cell line on SCID mice and established a subcutaneous xenograft model. Induction of TEAD2-DN by doxycycline administration *in vivo* induced a significant tumor growth inhibition (TGI) of 44% (*p* < 0,0001) when doxycycline was administered right after tumor cell implantation. When doxycycline was administered later, on already established tumors ranging from 171 to 414 mm3, the effect was a transient tumor stasis with a TGI of 50% (*p* < 0,0001) Fig. 2A-B.

**Fig. 3:**
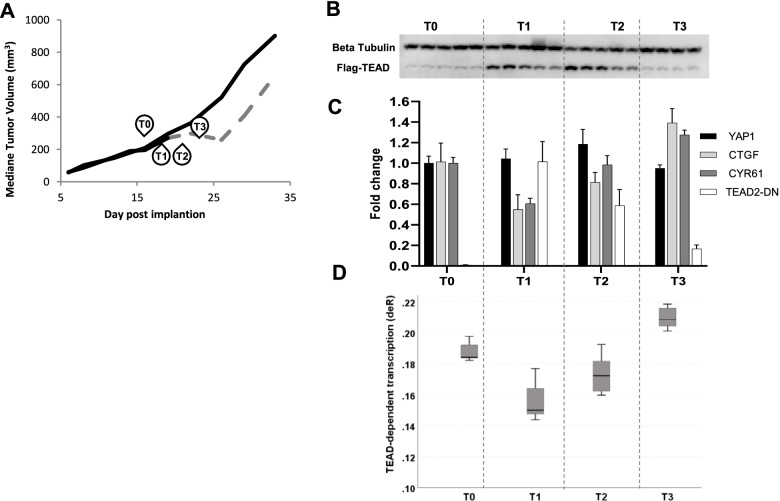
*In vivo* pharmacodynamic analysis of MSTO-211H-TEAD2-DN xenografts: **A** In this pharmacodynamic study, tumors were allowed to grow up to 300 mm.^3^ under glucose supplementation and were sampled just before doxycycline supplementation (time point 0, *n* = 5), 24 h (*n* = 5), 96 h (*n* = 5) and 216 h (*n* = 4) post doxycycline supplementation (time point 1, 2 & 3). **B** Western blot analysis of TEAD2-DN protein expression. Beta tubulin is used as a loading control. **C** mRNA expression by RT-qPCR. **D** TEAD-dependent transcription (deR)

In parallel, in an *in vivo* pharmacodynamic study, we evaluated biomarker modulation post-TEAD2-DN induction by doxycycline at 24 h, 96 h, and 216 h Fig. 3A. TEAD2-DN was highly expressed at 24 h and 96 h following induction by doxycycline. However, 216 h post continuous doxycycline administration, TEAD2-DN was no longer present Fig. 3B. The transient TEAD2-DN induction correlated with the inhibition of the downstream effectors CTGF (gene name CNN2) and CYR61(gene name CNN1) at the mRNA level for which a maximum inhibition was observed 24 h post doxycycline induction (45% and 39% respectively) and for which inhibition was lost at 216 h post doxycycline induction Fig. 3C. The transient inhibition was also detected at the protein level for CYR61 Fig. S2. Along the same lines, maximal YAP1 inhibition based on the YAP1 gene transcription signature score was observed 24 h post doxycycline induction and was gradually lost with no inhibition at all detectable at 216 h post doxycycline treatment Fig. 3D. We suspected that the transient nature of the TEAD2-DN effect stemmed from an escape mechanism and derivation of the cell population expressing TEAD2-DN *in vivo*. This assumption is supported by the loss of CYR61 and CTGF inhibition as well as the decrease in the YAP1 activation score observed at the very end of the *in vivo* tumor growth study 34 days post *in vivo* tumor cell implantation and 15 days post doxycycline treatment Fig. S3.

Nevertheless, the TEAD dominant negative approach showed that even transient downregulation of YAP1 and YAP1-TEAD target genes *in vivo* can lead to tumor growth inhibition in established tumors. To confirm these observations in a more stable setting over time, we decided to extend the *in vivo* target validation experiments to an orthogonal genetic model.

### YAP1 shRNA knockdown induced *in vivo* tumor regression and was specific for YAP1 activated MPM

**Fig. 4:**
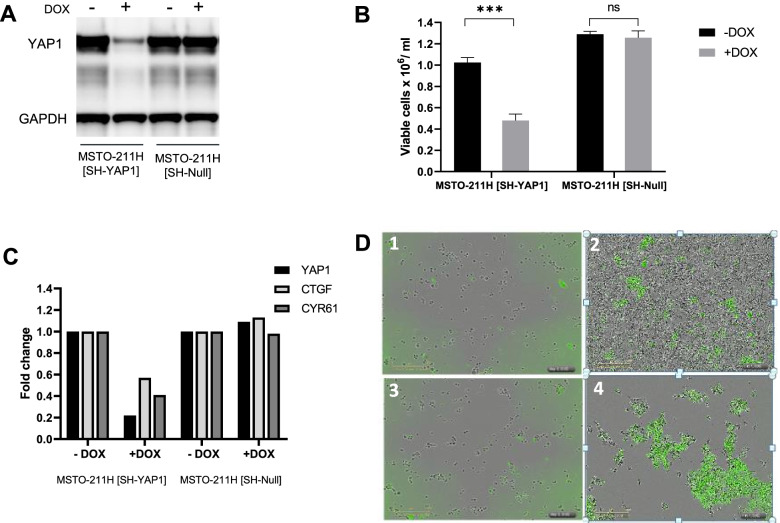
*In vitro* characterization of MSTO-211H-SH-YAP1 cells: **A** Western immunoblot analyses of YAP1 in the MSTO-211H-SH-YAP cell line. GAPDH was used as a loading control. **B** 2D cell viability of the MSTO-211H-SH-YAP cell line at 96 h post doxycycline induction. **C** RT- qPCR of YAP1 and the YAP1-TEAD downstream targets CTGF and CYR61 in the MSTO-211H-SH-YAP cell line 72 h post doxycycline induction. **D** Apoptosis analysis by Incucyte ®: Induction of apoptosis (green cells); no doxycycline at day 0 (1) and day 5 (2), addition of doxycycline at day 0 (3) and at day 5 (4)

We generated a construct expressing an shRNA against YAP1 behind a doxycycline-inducible promoter, stably transfected it into the MSTO-211H cell line and evaluated its effect *in vitro*. Doxycycline induction of YAP1shRNA led to 80% downregulation of YAP1 at the mRNA level and resulted in approximately 50% inhibition of tumor cell growth, 96 h post doxycycline induction Fig. 4A-B. YAP1 downregulation was accompanied by the inhibition of gene expression for the YAP1-TEAD target genes CYR61 (60%) and CTGF (45%) as determined by RT-qPCR. In addition, YAP1 knockdown led to significant induction of apoptosis Fig. 4C-D, and supplemental information for Incucyte® video.

**Fig. 5:**
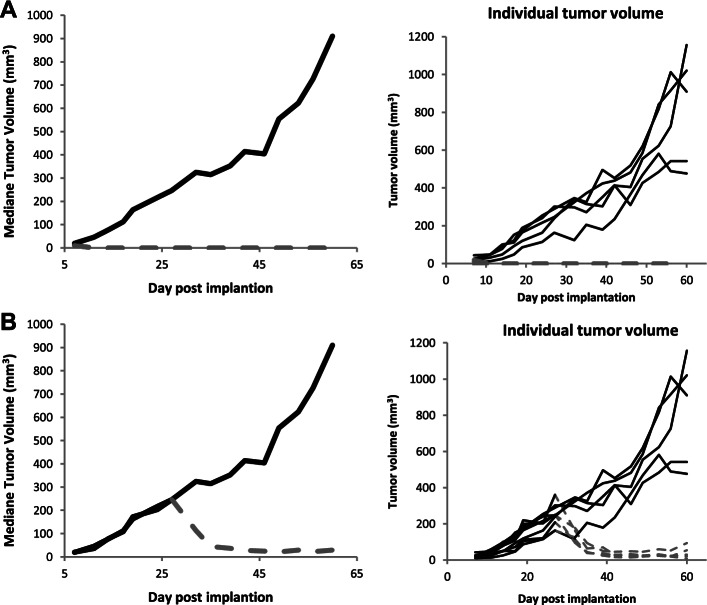
*In vivo* tumor growth of MSTO-211H-SH-YAP1 xenografts: **A** Mice were supplemented with either 5% glucose (black curve, *n* = 5) or doxycycline (dotted grey curve, *n* = 10) in drinking water at day 0 post MSTO-211H-SH-YAP cell line engraftment. **B** Mice were supplemented with 5% glucose in drinking water as soon as day 0 post MSTO-211H-SH-YAP cell line engraftment (black curve). At 27 days post MSTO-211H-SH-YAP cell line engraftment half of the mice (*n* = 5) bearing established tumors that ranged from 160 to 360 mm.^3^ were supplemented with doxycycline in drinking water (dotted grey curve curve)

We engrafted the MSTO-211H-SH-YAP1 cell line subcutaneously in a mouse model and evaluated its activity in a series of efficacy studies. Downregulation of YAP1 by the doxycycline inducible YAP1 shRNA prevented tumor initiation in 10 out of 10 mice Fig. 5A. Furthermore, knockdown of YAP1 following doxycycline supplementation in mice bearing established tumors with sizes ranging from 160 to 360 mm^3^, induced tumor regression in five out of five mice with a median regression of 80% as early as 12 days post doxycycline treatment and a maximum of 96% median regression at 39 days post doxycycline treatment Fig. 5B. Reinforcing the role of YAP1 in mesothelioma tumor growth, tumor regressions were also observed upon YAP1 knockdown in larger tumors, ranging from 476–1157 mm^3^. However, regressions were followed by a prompt regrowth of the tumors Fig S4.

**Fig. 6:**
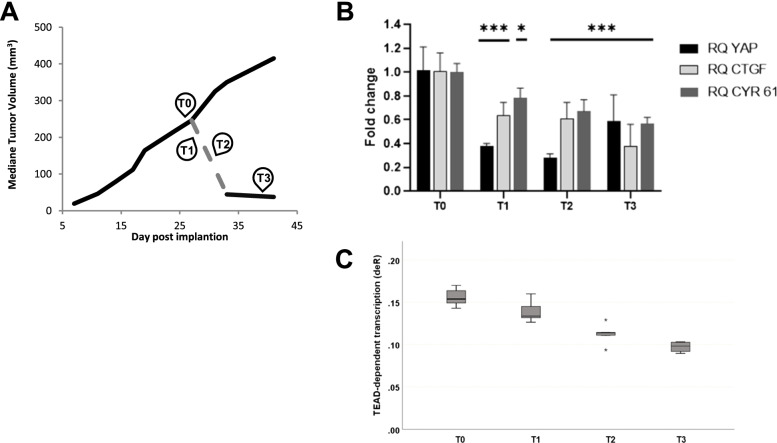
*In vivo* pharmacodynamic analysis of MSTO-211H-SH-YAP1 xenografts: **A** In this pharmacodynamic study, tumors were allowed to growth up to 300 mm.^3^ under glucose supplementation and were sampled just before doxycycline supplementation (time point 1, *n* = 5), 24 h (*n* = 5), 96 h (*n* = 5) and 216 h (*n* = 4) post doxycycline supplementation (time point 2 & 3) and 96 h post doxycycline induction followed by 6 days without doxycycline supplementation (time point 4). **B** mRNA expression by RT-qPCR. **C** TEAD-dependent transcription (deR)

We then evaluated biomarker modulation in an *in vivo* pharmacodynamic study 24 h and 96 h post doxycycline induction and at the time point of 96 h post doxycycline induction followed by 6 days without doxycycline treatment Fig. 6A. The last time point was intended to evaluate PD modulation upon tumor regrowth once doxycycline treatment was stopped, but surprisingly no tumor regrowth was observed after doxycycline removal**.** While YAP1 shRNA induction was associated with 62% and 72% knockdown of YAP1 mRNA 24 h and 96 h post doxycycline supplementation, respectively, 6 days post doxycycline removal, 41% inhibition of YAP1 mRNA was still present, possibly explaining the absence of tumor regrowth observed. Once again, YAP1 downregulation 24 h and 96 h post shRNA YAP1 induction was associated with a significant decrease of YAP1-TEAD dependent transcription as determined by RT-qPCR experiments for CTGF and CYR61, as well as by the inhibition of the signature scores. Furthermore, CTGF and CYR61 RNA biomarkers and the signature scores continued to drop even after doxycycline removal, which correlated with the observed continued inhibition of the tumor growth phenotype. Figure 6 B-C.

Finally, to demonstrate the specificity of the YAP1 shRNA effects, we evaluated the same doxycycline inducible YAP1 shRNA knockdown construct in a Hippo pathway independent colon cancer model, HCT116. In cell culture, doxycycline treatment led to 87% knockdown of YAP1 mRNA and downregulated the expression of YAP1-TEAD target genes CYR61 and CTGF, but contrary to what we observed with the MSTO-211H cell model, we did not detect any effect on tumor cell growth for HCT-116, indicating that HCT116 cell growth does not depend on YAP1 Fig. S5 A-B-C. In full alignment with these *in vitro* data, the subsequent *in vivo* studies with YAP1 shRNA HCT-116 xenograft models demonstrated that YAP1 knockdown had no effect on tumor growth and prevented neither tumor initiation in 6 out of 6 mice nor did it induce tumor regression on already established tumors (165 to 561 mm^3^) in 6 out of 6 mice tested Fig. S5 D-E. These data strongly suggest that the growth inhibitory effect observed with YAP1 KD in the MPM MSTO-211H *in vivo* model was specific and not a general cytotoxicity phenomenon.

Interestingly, tumoral regressions achieving 96% were obtained upon doxycycline treatment in mice bearing MSTO-211H-SH-YAP1, but a tumor escape was noticed following extended exposure to YAP1 shRNA Fig. S4**. To further investigate this tumor escape, a new batch of mice was engrafted with one of the re-growing tumors and evaluated for sensitivity to YAP1 shRNA expression upon doxycycline supplementation. We observed that shRNA induction still led to the downregulation of YAP1 protein levels as determined by Western blot in this long-term treatment model. However, we did not detect any effect on tumor growth anymore, indicating that this tumor model had become independent from exclusive regulation by YAP1 Fig. S6. We consider this model as an *in vivo* model of acquired resistance to YAP1 inhibition and believe that it merits further comprehensive analyses as it can be a valuable tool for studying escape of treatment with YAP1 pathway inhibitors. The mechanism of resistance in this tumor model will be the subject of our future studies.

### Pharmacological inhibition by allosteric TEAD inhibitor K-975 mimics genetic studies in MPM *in vivo* models

Recently, therapeutic approaches directed at targeting aberrant YAP1 activation in tumors have advanced. In particular, allosteric inhibitors blocking TEAD palmitoylation and thereby inhibiting YAP1-TEAD-dependent gene transcription have been developed [15, 38–42]. K-975 is one of these allosteric TEAD inhibitors [15], and we compared the activity of this compound to our genetic studies *in vivo*.

**Fig. 7:**
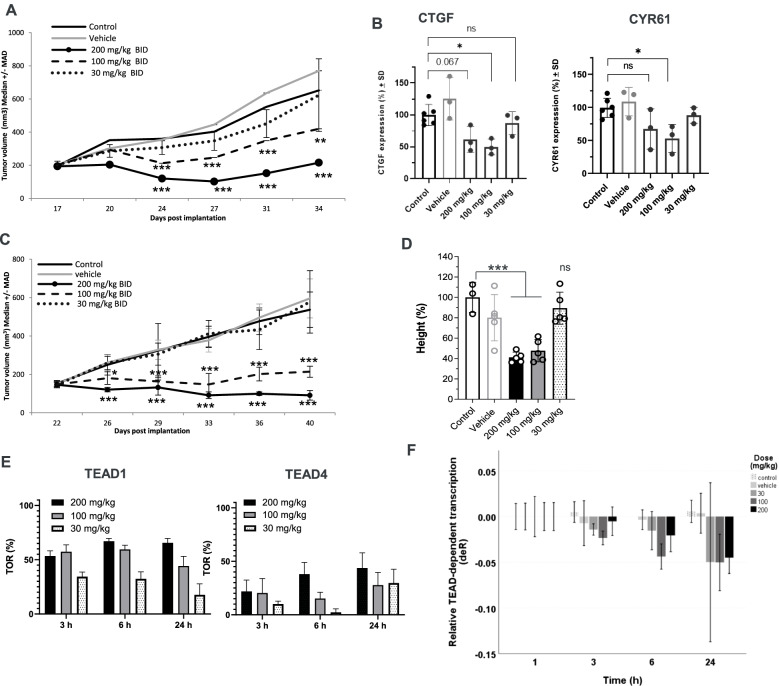
*In vivo* efficacy and biomarker modulation of K-975 in MSTO-211H and NCI-H226 xenografts: **A** *In vivo* efficacy of K-975 in MSTO-211H tumor bearing mice. **B** CTGF and CYR61 protein levels in tumors as determined by WES, 24 h post single K-975 administration in MSTO-211H tumor bearing mice. **C** *In vivo* efficacy of K-975 in NCI-H226 tumor bearing mice. **D** CYR61 protein levels in tumors by WES 24 h post single K-975 administration in NCI-H226 tumor bearing mice (*n* = 3). **E** Target occupancy ratio: TEAD1/4 covalent TEAD binding (see supplementary methods). **F** TEAD-dependent transcription post single K-975 administration in NCI-H226 tumor bearing mice (deR; error bars represent 95% confidence limits)

We first assessed the *in vivo* efficacy of K-975 in the MSTO-211H xenograft model. Robust antitumor activity was observed with 49% median regression after treatment with K-975 at the highest dose tested of 200 mg/kg twice a day (BID) for 18 consecutive days. The regressions were transient since tumors started to regrow under treatment. At lower doses, 100 mg/kg BID resulted in tumor growth delay, and K-975 was inactive at 30 mg/kg BID Fig. 7A. In accordance with the level of *in vivo* antitumor activity, K-975 was able to decrease CTGF and CYR61 protein levels at the active doses of 200 and 100 mg/kg, but not at the inactive dose of 30 mg/kg, 24 h after single administration in a corresponding pharmacodynamic study Fig. 7B.

We then continued the evaluation of K-975 in the NCI-H226 xenograft model, an MPM model harboring an NF2 deletion. Robust antitumor activity was also observed in this model with 34% median regression of tumors after 200 mg/kg BID treatment for 18 consecutive days. The treatment dose of 100 mg/kg BID was also active, but with tumor stasis, and no efficacy was obtained at the dose of 30 mg/kg BID Fig. 7C. In line with the NCI-H226 *in vivo* efficacy study and similar to what we had previously seen in the MSTO-211H MPM xenograft model, K-975 was able to modulate CYR61 protein levels 24 h after a single administration at the active doses of 200 and 100 mg/kg, but not at the inactive dose of 30 mg/kg in the corresponding pharmacodynamic study Fig. 7D. Since K-975 is a covalent inhibitor forming a stable bond with the cysteine at the entry of the TEAD allosteric lipid pocket [15, 38, 40, 43] we also evaluated the degree of TEAD target occupancy by this molecule by determining the Target Occupancy Ratio (TOR) [44]. This study aimed to relate the on-target binding of TEAD with PD biomarker modulation and efficacy results. Out of the four members of the TEAD protein family, the NCI-H226 model predominantly expresses TEAD1 and TEAD4, so TOR was determined for these two TEAD proteins. Significant time and dose effects were observed for K-975 on TOR measured on TEAD1 and TEAD4, with a maximal target engagement of 67% on TEAD1 and a lower TOR of 38% on TEAD4 at 6 h after a single administration of 200 mg/kg Fig. 7E. Regarding biomarker modulation, significant dose and time-dependent inhibition were observed for both the YAP1-TEAD transcription score Fig. 7F and the downregulation of CYR61 protein levels (60% inhibition), 24 h after a single administration of 200 mg/kg. Hence, based on this data, 67% TOR on TEAD1, 30–40% decrease in the YAP1-TEAD transcription score, and 60% downregulation of CYR61 protein levels are sufficient to achieve tumor regression in the MPM NCI-H226 model.

In summary, the TEAD allosteric inhibitor K-975 led to the regression of established MPM tumors *in vivo*. K-975 efficacy and dose–response could be associated with the degree of TEAD target occupancy and with the modulation of YAP1 biomarkers such as the YAP1-TEAD dependent gene transcription score and CYR61 protein levels. While the *in vivo* tumor growth inhibition and biomarker modulation effects of the small molecule compound qualitatively compared to the effects we obtained with the YAP1 shRNA, the maximal tumor growth inhibition for K-975 was inferior to what we had observed with the genetic YAP1 shRNA knockdown. Whether this is due to the pharmacological properties of the K-975 compound or to its mechanism of action (TEAD binding versus YAP1 downregulation) will need to be further explored.

## Discussion

Here we report for the first time a complete target validation study based on a multi-functional approach in which we clearly establish the importance of YAP1-TEAD for tumor maintenance in MPM. Using two different inducible genetic systems, TEAD2-DN and YAP1 shRNA in the Hippo deleted MPM cell line MSTO-211H as a mouse tumor xenograft model, we show that the inhibition of YAP1-TEAD not only prevented the initiation of tumors but is sufficient for blocking the growth of fully established tumors *in vivo*. The observed effects were specific, as the *in vivo* tumor growth inhibition in response to YAP1 inhibition was associated with Hippo-YAP1 pathway biomarker modulation and was not observed in the Hippo-YAP1-independent HCT116 colorectal cancer xenograft model. In agreement with the genetic studies, using a pharmacological approach with K-975, a recently published allosteric inhibitor of TEAD palmitoylation, we also demonstrated antitumor activity *in vivo* in two different YAP1 activated MPM models [15]. Taken together, these data strongly suggests that YAP1-TEAD is a significant driver of tumor maintenance in Hippo-deregulated and YAP1 activated MPM tumors and highlights the attractiveness of this pathway for cancer therapy.

Current treatment options for MPM patients are poor and most patients with mesothelioma are not offered surgery because of the extent of disease, advanced age, and poor performance status. For a long time, the gold standard of treatment has been the combination of cisplatin and pemetrexed [1] until the FDA approved an immunotherapy combination treatment with nivolumab and ipilimumab in MPM as first line treatment in October 2020. However, treatment benefit remains limited, and no targeted therapy is yet available. According to literature references and TCGA data analysis, Hippo-YAP1 pathway deletions are common in MPM patients and are present in approximately 50% of all patients [12–14] (TCGA; US National Cancer Institute, https://www.cancer.gov). Here, however, we purport that there is an even higher prevalence of Hippo-YAP1 pathway regulator alterations (estimated to 80–90%) in MPM after our detailed examination of MPM patient tumors from the TCGA database. In addition, patients with higher YAP1-TEAD transcription scores had significantly shorter overall survival than patients with lower scores [34]. Our data suggest that MPM is an indication primarily driven by YAP1-TEAD activation and could be highly responsive to YAP1-TEAD pathway inhibitors.

Other tumor indications with Hippo-YAP1 pathway alterations have also been reported, including tumors with YAP1 amplifications such as cervical cancer or tumors with NF2 deletions such as renal cell carcinoma and schwannomas meningiomas [34, 41, 45]. When looking at tumors with aberrant YAP1 activation (TEAD-activity score higher than the 90^th^ percentile) for which a genetic link cannot always be provided, the list of indications is even longer and the examples include lung, liver, skin, pancreas, breast, uterus, prostate, head and neck cancers and gliomas (reviewed in [9, 46]).

Finally, YAP1 activation is emerging as a mechanism of resistance to cancer therapies. It has been reported to occur in response to different types of therapies, ranging from targeted therapies after receptor tyrosine kinase and MAPK pathway inhibition to chemo- and even immune therapies (reviewed in [47]). Whether YAP1-TEAD are drivers of tumor growth in some of these indications and whether YAP1 inhibition will suffice for *in vivo* activity on tumor maintenance remains to be established and will require similar genetic and pharmacological studies as shown here.

Pharmacological inhibitors of YAP1-TEAD activity may be helpful beyond tumors with Hippo-YAP1 alterations and could be employed for a broader set of combination treatment strategies. Drug discovery efforts for the identification of YAP1-TEAD inhibitors have indeed progressed over recent years (reviewed in [48]). In particular, compounds targeting the allosteric lipid pocket of TEAD proteins have been put forward with a set of molecules in preclinical development [15, 39], and the first compounds from Vivace Therapeutics and Ikena Oncology entered clinical trials last year and early this year, respectively ( [41], NCT04665206, NCT05228015). The K-975 molecule we used in the studies presented here is one of the described TEAD palmitoylation inhibitors. We confirmed that K-975 inhibited MPM tumor growth *in vivo* and modulated YAP1-TEAD dependent gene transcription, similar to what we had observed when using a genetic knockdown of YAP1. However, the *in vivo* activity obtained with K-975 was below the activity we had obtained with YAP1 knockdown. This may simply be due to the mechanism of action: pharmacological inhibition of TEAD-YAP1 by K-975 may be less effective than partial removal of the YAP1 protein from the system by shRNA. However, it may also be due to the profile of K-975, for which we detected a higher target occupancy of TEAD1 compared to TEAD4 in our assays. We can speculate that equal and more complete target occupancy of both TEAD1 and TEAD4 is required in NCI-H266 to drive a more potent antitumor activity, comparable to the genetic knockdown of YAP1.

Further pharmacological studies with other TEAD binding molecules will be needed to determine the best profile for YAP1-TEAD inhibition in the different tumor types. Whether a pan-TEAD inhibitor would be superior to a TEAD isoform-specific compound and how their safety profiles would compare will also need to be determined. Direct targeting of YAP1 could also be an alternative to TEAD allosteric compounds, as is being investigated by Ionis Pharmaceuticals, who have recently started Phase I clinical trials using an antisense oligonucleotide (ASO) targeting YAP1 mRNA (NCT04659096). Finally, different tumor types may respond better to one or another mechanism of inhibition. At this point, we have just started to understand the high potential of inhibiting the Hippo-YAP1 pathway for cancer therapy. Additional genetic and pharmacological studies in different tumor models, including studies in syngeneic mouse models with an intact immune system, will be needed to fully understand the best way of targeting this pathway alone or in combination with other agents.

## Supplementary Information

**Additional file 1.**

**Additional file 2.**

**Additional file 3.**

## Data Availability

The RNA-sequencing data generated and analysed during the current study are available in the GEO repository, https://www.ncbi.nlm.nih.gov/geo/query/acc.cgi?acc=GSE196672 https://www.ncbi.nlm.nih.gov/geo/query/acc.cgi?acc=GSE196691 https://www.ncbi.nlm.nih.gov/geo/query/acc.cgi?acc=GSE196726 https://www.ncbi.nlm.nih.gov/geo/query/acc.cgi?acc=GSE196714
