# Supplementary material for: YAP1 is essential for malignant mesothelioma tumor maintenance
Source: BMC Cancer. 2022 Jun 10;22:639. doi: 10.1186/s12885-022-09686-y (PMC9188206; doi:10.1186/s12885-022-09686-y)
Supplement: Supplementary file 3 — Additional file 3. [file 12885_2022_9686_MOESM3_ESM.docx]

**Supplemental Information**

YAP1 is Essential for Malignant Mesothelioma Tumor Maintenance

Loreley Calvet^1 *^, Odette Dos-Santos^2^, Emmanuel Spanakis^3^, Véronique Jean-Baptiste^2^, Jean-Christophe Le Bail^2^, Armelle Buzy^5^, Pascal Paul^5^, Christophe Henry^2^, Sandrine Valence^3^, Colette Dib^3^, Jack Pollard^4^, Sukhvinder Sidhu^1^, Jürgen Moll^2^, Laurent Debussche^1,2^, and Iris Valtingojer^2^

^1^Department of Oncology, In Vivo Pharmacology, Sanofi Research Center, Vitry-sur-Seine, France

^2^Department of Oncology, Molecular Oncology, Sanofi Research Center, Vitry-sur-Seine, France

^3^Department of Oncology, Precision Medicine Oncology, Sanofi Research Center, Vitry-sur-Seine, France

^4^Department of Oncology, Precision Medicine Oncology, Sanofi Research Center, Cambridge, US

^5^Department of Translational Sciences, Sanofi Research Center, Chilly Mazarin, France

^*^Corresponding author **email address**: [loreley.calvet@sanofi.com](mailto:loreley.calvet@sanofi.com)

**Supplementary Methods:**

- **DNA sequences**
- Sh-Yap

5’GAACAATGACGACCAATAGCTCTCGAGAGCTATTGGTCGTCATTGTTCTTTTT3’

- Human TEAD2-DN (Ser112-Asp447)

TCAAGGGAAATCCAGTCCAAGTTGAAGGACCAGGTTTCCAAGGACAAGGCTTTCCAGACAATGGCAACCATGTCCTCTGCCCAGCTCATCTCCGCGCCTTCTCTGCAGGCCAAACTGGGTCCCACTGGTCCTCAGGCCTCTGAGCTTTTCCAGTTTTGGTCTGGAGGATCTGGGCCCCCCTGGAATGTTCCAGATGTGAAGCCATTCTCACAGACACCGTTCACCTTGTCACTGACTCCCCCATCTACTGACCTCCCAGGGTACGAGCCCCCCCAAGCCCTCTCACCCCTGCCCCCACCTACCCCATCGCCCCCAGCCTGGCAGGCTCGGGGCCTGGGCACCGCCCGGTTGCAGCTGGTAGAGTTCTCAGCCTTCGTGGAACCGCCAGATGCAGTTGATTCTTACCAGAGGCACCTGTTCGTGCACATCAGCCAGCACTGCCCCAGCCCCGGAGCGCCGCCGCTCGAGAGTGTGGACGTCCGGCAGATCTACGACAAATTCCCTGAGAAAAAGGGTGGCCTCCGAGAGCTATATGATCGTGGTCCTCCACATGCCTTCTTCCTGGTCAAGTTCTGGGCGGACCTGAACTGGGGCCCAAGTGGTGAGGAGGCAGGGGCCGGTGGCAGCATCAGCAGTGGTGGCTTCTACGGAGTGAGCAGCCAGTATGAGAGCCTGGAACACATGACCCTCACCTGTTCCTCCAAGGTCTGCTCTTTTGGCAAGCAGGTGGTGGAGAAGGTGGAGACGGAACGGGCCCAGCTGGAGGACGGCAGATTTGTGTACCGCCTGCTGCGCTCGCCCATGTGCGAGTACCTGGTGAATTTCTTGCACAAGTTGCGGCAGCTGCCTGAGCGATACATGATGAACAGCGTCCTGGAAAACTTCACCATCCTCCAGGTGGTGACAAACAGAGACACCCAGGAACTGCTGCTCTGCACCGCCTATGTCTTCGAGGTCTCCACCAGCGAGCGTGGGGCCCAGCATCACATTTACCGCCTGGTCAGGGACTAG

- **Elisa assay**

Tumor lysates were diluted 1/500 in cell lysis buffer. A minimum of four biologic replicates were analyzed for each model. In the reading plate, standards and samples are run in duplicate. hCYR61 was assessed using the human Cyr61 quantikine ELISA kit (R&D systems DCYR10). Diluted lysates were processed and analyzed as described in the manufacturer’s instructions. Absorbance values were read using a microplate reader set to 450 nm.

- **TEAD1 & TEAD4 Target Occupancy Ratio (TOR) measurement in tumor xenografts**

For TOR measurement by targeted Mass Spectrometry, 60 µg of total proteins from tumor lysates were diluted in Laemmli buffer and separated by SDS-PAGE under reducing conditions. A gel band covering the 50 kDa area, corresponding to TEAD proteins molecular weight, was excised, reduced with DTT, alkylated with iodoacetamide and in-gel digested with trypsin. Peptides were extracted with 50 mM ammonium bicarbonate and 50 % acetonitrile in 0.2 % formic acid, dried by evaporation in a speed-vac concentrator and resuspended in 60 μl of 0.2% formic acid. Samples were spiked with four Heavy isotopic AQUA standards peptides at 10 fmol each, provided by ThermoFisher Scientific and subjected to targeted Liquid Chromatography/Mass spectrometry (LC/MS) analysis. Analyses were performed using a nano-ACQUITY Ultra-Performance LC system (Waters, Milford, MA) coupled to an Orbitrap Fusion Tribrid mass spectrometer (Thermo Fisher Scientific, San Jose, CA). LC separation was performed with a trapping column (nano-Acquity Symmetry C18, 100 Å, 5 μm, 180 μm x 20 mm) at 15 μl/min flow rate and an analytical column (nano-Acquity BEH C18, 130 Å, 1.7 μm, 75 μm x 250 mm) directly coupled to the ion source. The mobile phases for LC separation were 0.2% (v/v) formic acid in LC-MS grade water (solvent A) and 0.2% (v/v) formic acid in acetonitrile (solvent B). Peptides were separated at a 300 nl/min constant flow rate with a linear gradient of 5-85% solvent B for 35min. LC/MS-MS-based assays were adapted from [1]. In brief, the Precursor Reaction Monitoring (PRM) acquisition mode was used to monitor the light and heavy TEAD1 and TEAD4 central pocket cysteine peptides, SPMCEYMINFIHK and SPLCEYMINFIHK, respectively and TEAD1 and TEAD4 normalization peptides, GPQNAFFLVK and GPSNAFFLVK, respectively. Fragmentation was performed in HCD fragmentation cell (collision energy at 27%), with isolation of precursor ions in the quadrupole. The detection was performed in the orbitrap with an Automatic Gain Control (AGC) of 20e4 target value and a 150 ms maximum injection time. Resolution was set to 30k at m/z 200. Each sample was injected twice (technical replicate). The data were processed using Skyline version 3.6 (University of Washington; Seattle, WA). TEAD1 and TEAD4 TOR measurements for each treated sample were calculated according to the following formula:

$$TOR=\left[ 1-\frac{Peak area treated Cysteine peptide}{Peak area Vehicle Average Cysteine Peptides}x\frac{Peak area Vehicle Average Normalisation peptides}{Peak area treated Normalisation Peptide} \right]*100$$

**[1]** Patricelli M, Janes MR, Li L-S, Hansen R, Peters U, Kessler LV, Chen Y, Kucharski JM, Feng J, Ely T, Chen JH, Firdaus SJ, Babbar A, Ren P, Liu Y. Selective Inhibition of Oncogenic KRAS Output with Small Molecules Targeting the Inactive State. Cancer Discover. 2016, 6(3):316-329.

**Supplementary Data**

- **List of 249 positive YAP1/TAZ-TEAD effector genes.**

AASS, ABAT, ACAT2, ADAMTS1, ADM, ADRB2, AMOT, ANXA3, ARHGAP11A, ARHGDIB, AURKB, AVPI1, AXL, AZIN1, B4GALT4, BCAT1, BIRC5, BTG3, C4BPB, CAP2, CAV1, CAVIN1, CCBE1, CCDC80, CCN1, CCN2, CDC25A, CDC6, CDCA3, CDCA4, CDCA5, CDCA8, CDH4, CDK2, CDK6, CDV3, CENPA, CENPI, CENPM, CENPN, CHRNB1, CHST13, CKS2, CLDN1, CLIC3, CNN3, COBL, COL8A1, COTL1, CPA4, CRIM1, CRY1, CTH, CXCL1, CYTH3, DAPK1, DCLRE1B, DDAH1, DHCR7, DHFR, DIAPH3, DKK1, DLL1, DONSON, DUSP14, DUT, EBP, EIF2AK3, EMG1, EPHA2, EPS8L2, ESM1, ETS1, EXO1, EXOSC2, F3, FAHD2A, FAM83D, FANCA, FAT4, FDPS, FEN1, FMR1, FST, FSTL1, FSTL3, GADD45A, GADD45B, GINS1, GPC6, GPR176, GPRC5A, GPRC5B, GRAMD2B, HASPIN, HEG1, HEXB, HPS5, HSPB11, IDI1, IGFBP7, IKBIP, IL6, ITGB2, JDP2, JPH2, KPNA2, KRT8, KRT80, LCA5, LHFPL6, LMCD1, LMNB2, LRP8, LRRFIP2, LSM5, LYPD6, LYRM1, MAD2L1, MAP6D1, MATN2, MATN3, MCM10, MCM2, MCM5, MDC1, METRNL, MICB, MID1, MRPL33, MSRB3, MVD, MXRA7, NCAPD3, NEDD4, NEDD4L, NEK2, NEXN, NFIB, NNMT, NOC3L, NTN4, NUAK1, NUAK2, NUDCD1, NUP107, NUP37, OGFRL1, OLFML3, OLR1, OXCT1, PAK2, PCBD1, PCNA, PDLIM2, PDZD2, PEPD, PHLPP1, PKMYT1, PKP2, PKP4, PLCE1, PLEKHA7, PLK2, PLOD2, PPIH, PRPS1, PRPS2, PRSS23, PSG2, PSG6, PSG7, PSG9, PVR, PXMP2, QDPR, QKI, RAB11FIP1, RAB32, RACGAP1, RBM24, RBMS2, RCN2, RFC4, RND3, RNF144B, ROR1, RPS24, SCD5, SCML1, SDC2, SEC14L1, SGK1, SGMS2, SGTB, SH3RF1, SHCBP1, SKP2, SLC25A23, SLC25A3, SLC38A5, SLC3A2, SLC7A1, SLC7A5, SMPD4, SNAPC1, SNX24, SORT1, SPAG1, SPATA5, STK3, STX11, STXBP6, SUSD2, SUV39H1, SYDE2, TACC3, TAGLN, TEAD1, TEAD4, TENT5B, TGM2, THBS1, TK1, TMEM139, TMEM160, TNFAIP3, TNFRSF12A, TNNC1, TPM1, TPX2, TRIP13, TSPAN2, TTF2, TUBB6, TUFT1, TYMS, UAP1, UBE2C, UGCG, UHRF1, VKORC1L1, WWC1, WWC2, YAP1, ZBED2, ZDHHC18, ZNF488, ZNF704.

- **List of 233 negative YAP1/WWTR1-TEAD effector genes**

AASDH, ABCA1, ABCC5, ABI3BP, ABLIM3, ACADVL, ACOT11, ACOX2, ACSL5, ADAM28, AGL, AGPAT4, ALDH3A2, ANKRD12, ANKRD22, ANKRD29, ANKRD42, ANTXR2, APBB3, ARAP3, ARHGEF2, ASF1A, ATP7A, ATXN1, BCL11B, BHLHE41, BMF, CA2, CASP1, CBR3, CCNG2, CDC42EP4, CDK1, CEBPB, CELSR3, CLCN3, CLDN4, COL6A1, COL6A2, CPE, CRABP2, CROT, CSRNP2, CSTA, CTNNBIP1, CTSB, CTSK, CXXC5, CYP1B1, CYP27C1, DDR1, DEDD2, DHX32, DIAPH2, DSC2, DSG3, DUSP6, DYNC2LI1, ELN, EPS8L3, ERAP2, FAM102A, FAM117B, FAM83B, FAM89B, FERMT1, FKBP2, FOS, FTH1, FXYD3, GDPD1, GOLGA5, GOLPH3L, GPNMB, GPRC5C, GRB10, GSN, HAS3, HBP1, HDAC1, HDHD2, HEY1, HOXA5, IFI44, IGSF3, IGSF9, INTS3, IRAK2, IRF9, IRX5, ITGA2, KCNMA1, KCNMB3, KCNN4, KIFAP3, KLF10, KLF13, KLHL3, KLK11, KRCC1, KRIT1, KRTDAP, LMTK3, LRP10, LTBP4, LXN, LYPD3, MALL, MANSC1, MAPK13, MARCKSL1, MFSD1, MFSD5, MGST2, MGST3, MLLT11, MLPH, MMP13, MSX2, MTMR11, MTMR9, MTSS1, MYO1A, NAGK, NAPEPLD, NCOA3, NFIL3, NPAS2, NRIP1, OAS1, OAS2, OASL, OFD1, OSBPL7, OTUB2, OVOL1, PAG1, PAK1, PCDHB2, PCDHB9, PCGF3, PCMTD2, PERP, PHF21A, PIK3C2B, PIK3R1, PIK3R2, PIK3R3, PIP4P2, PJA2, PKIA, PLA2G4C, PNRC1, PPP1R11, PRRX2, PTPRE, PYGB, RAC2, RALGPS1, RAPGEFL1, RBM23, RBM45, RBM47, RBP1, REEP6, RGL2, RGS17, RHOC, S100A14, SAMD9, SEC14L2, SECISBP2, SH3PXD2A, SH3TC1, SHROOM2, SLC14A1, SLC1A2, SLC30A9, SLC35C1, SLC37A2, SLC39A11, SLFN5, SLITRK6, SLK, SMOC1, SNCG, SP1, SPIRE2, SPRY4, SQSTM1, SRD5A3, SSPN, STMN3, STX1A, TBX3, TCF25, TDO2, TET2, TFF1, TLR3, TMC4, TMC7, TMEM140, TMEM144, TMEM45B, TP53INP1, TP63, TPD52L1, TRAPPC6B, TRIB1, TRIB2, TRIM13, TRIM31, TRIM38, TRIOBP, TRIP11, TSC22D1, TSPAN1, TTC17, TTLL3, TUBB3, UBC, ULK1, VAMP8, VGF, VPS52, VSNL1, WDR13, ZCWPW1, ZNF292, ZNF467, ZNF75D, ZSWIM7.

- **List of the 85 genes interrogated for mutations and copy-number aberrations**

**Hippo core pathway or related genes:**

AKT1, **AMOTL1**, **AMOTL2**, **ANKHD1**, APBA1, ASCL1, **BAP1**, BTRC, CCDC85C, CDK1, **CEBPA**, **CHRNA3**, **CSNK1D**, CSNK1E, CTNNA1, **ERBB4**, ESR1, **FBXW7**, GBP1, HNRNPU, **ITCH**, **LATS1**, **LATS2**, MALAT1, **MAP2K1**, **MAPK1**, **MAPK12**, MAPK14, **MAPK3**, **MAPK8**, MAPK9, MOB1A, MOB1B, **NF2**, **NR3C1**, **PML**, PPP1CA, **PPP2CA**, PRKCZ, **PRRT2**, **PTPN14**, RCOR1, RUNX3, **SAV1**, **SKP1**, **SLC9A3R1**, **SMAD3**, **SRSF1**, STK3, **STK38L**, STK4, SUMO1, SUZ12, **TCF7L2**, TEAD1, TEAD2, **TEAD3**, TEAD4, **TET1**, TJP2, TNFSF10, **TP53**, UBC, **VGLL1**, VGLL3, VGLL4, **WBP2**, **WWOX**, **WWTR1**, YAP1, YES1, YWHAH, **YWHAQ**, YWHAZ

**Other genes:**

ALB, **CDKN2A**, **CDKN2B**, EGFR, EIF1AX, **GNA11**, GNAQ, **KRAS**, **NRG1**, **RB1**, **SF3B1**

Shown in bold, we identified alterations in 38 of the 74 Hippo-related genes, and 7 of the other 11 genes, in TCGA MESO cohort.

- **Supplementary Incucyte**® **video**

Apoptosis analysis by Incucyte®:

- Video1= No doxycycline from day 0 to 5
- Video2= Addition of doxycycline from day 0 to 5
- **Figure S1**

**A:**
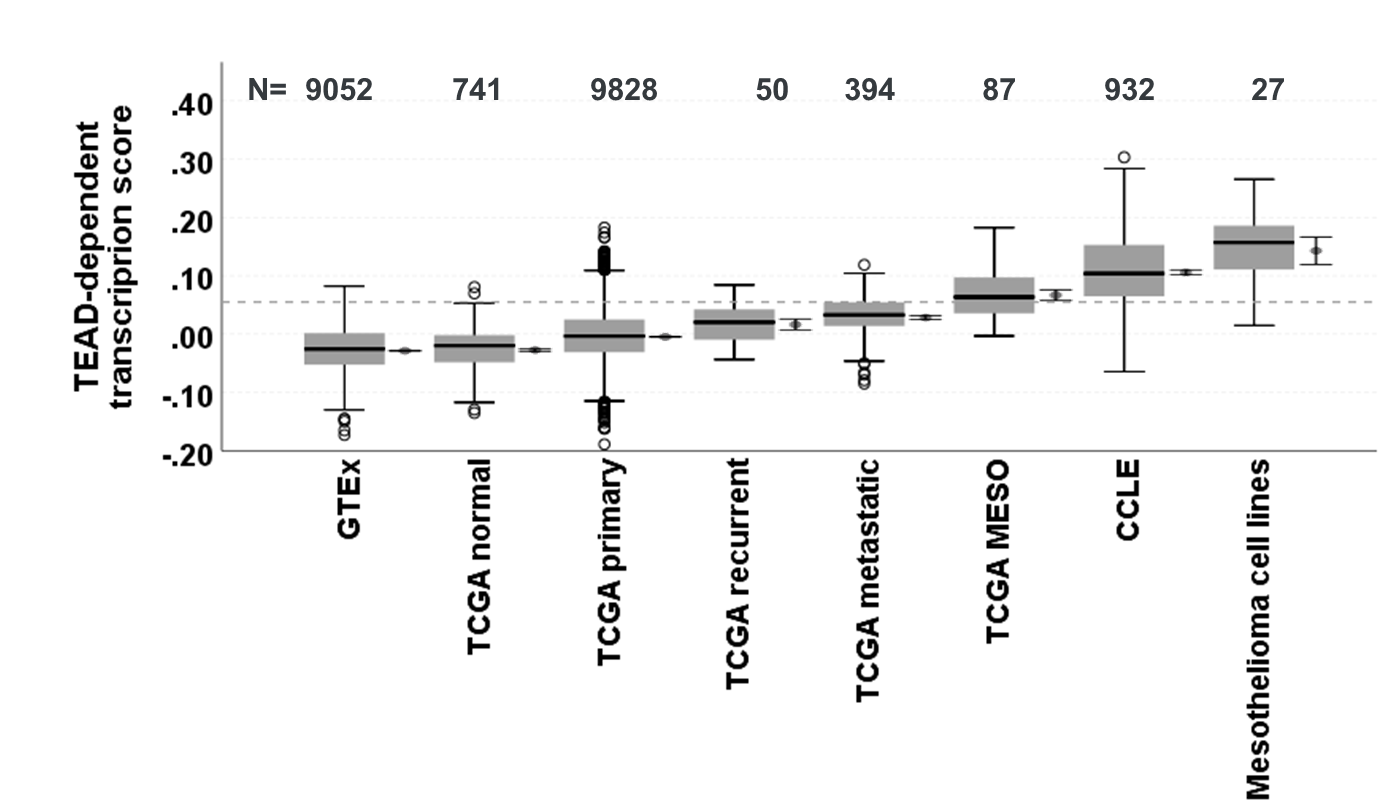


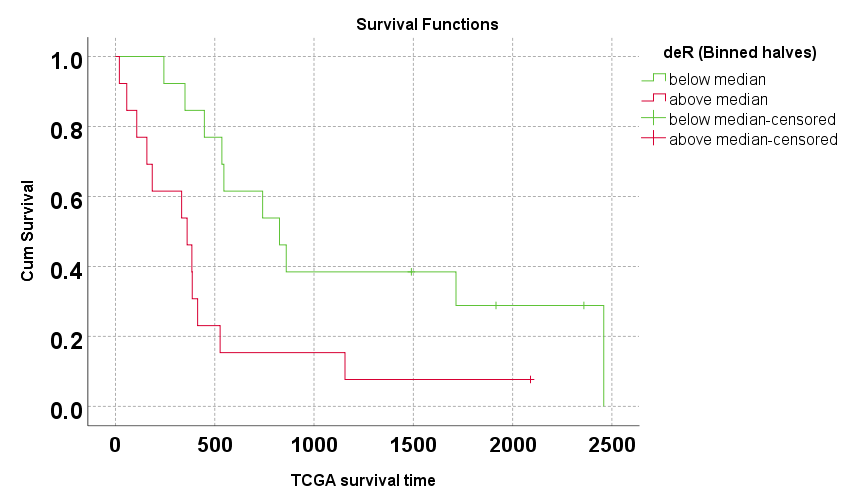

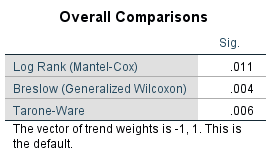

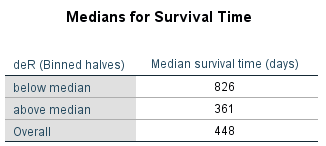


**B:**

**
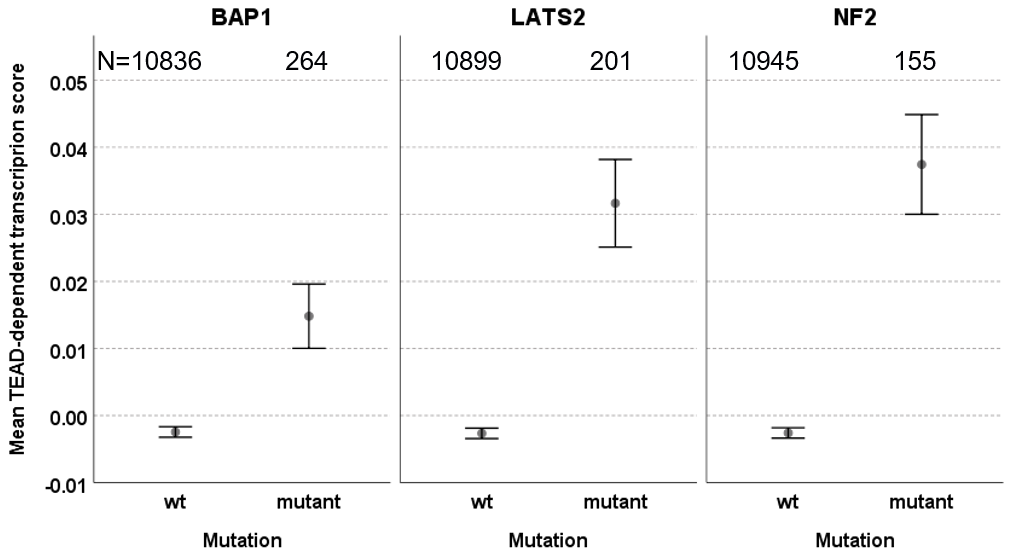
**

**C:**

**
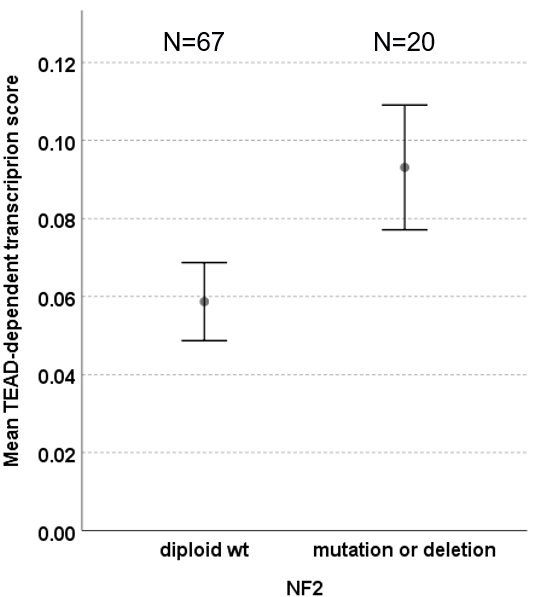
**

**Figure S1:** Distributions of the TEAD-dependent transcription score. **A**. In normal tissues, tumors, and cell lines. The score distribution moves towards higher values in tumors (TCGA v8, 02/15/2019) compared to normal tissues (GTEx Portal v4, 02/15/2019). Cell lines (CCLE v7, 02/15/2019) attain much higher scores, and mesothelioma cell lines are among the top scorers. **B**. Means in BAP1, NF2, or LATS2 mutated TCGA primary tumors compared to wild type. **C**. Means in TCGA MESO tumors with NF2 mutation or deletion compared to tumors with diploid, wildtype NF2. Error bars represent 95% confidence limits.

- **Table S1**
-

**Table S1: Malignant pleural mesothelioma cell lines characterization**

The score **deR** (**d**ifference of **e**ffector **R**anks) represents the activity of TEAD-family transcription factors. It is computed in single samples as the difference between the mean percentile ranks of the RNA-sequencing levels of some 500 positive and negative effectors of TEAD. **FPKM** (**F**ragments **P**er **K**ilobase per **M**illion mapped reads). The deR (difference in effector ranks) value is our YAP1-TEAD-activity signature score based on YAP1-TEAD-dependent transcription measurements.

- **Figure S2**


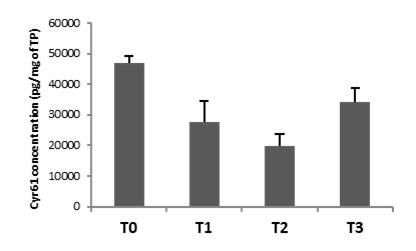


**Figure S2:** **hCyr61 proteomic assessment by Elisa**

Cyr61 proteomic evaluation just before doxycycline supplementation (time point 0, n=5), or 24h (n=5), 96h (n=5) and 216 (n=4) post doxycycline supplementation (time point 1, 2 & 3).

- **Figure S3**


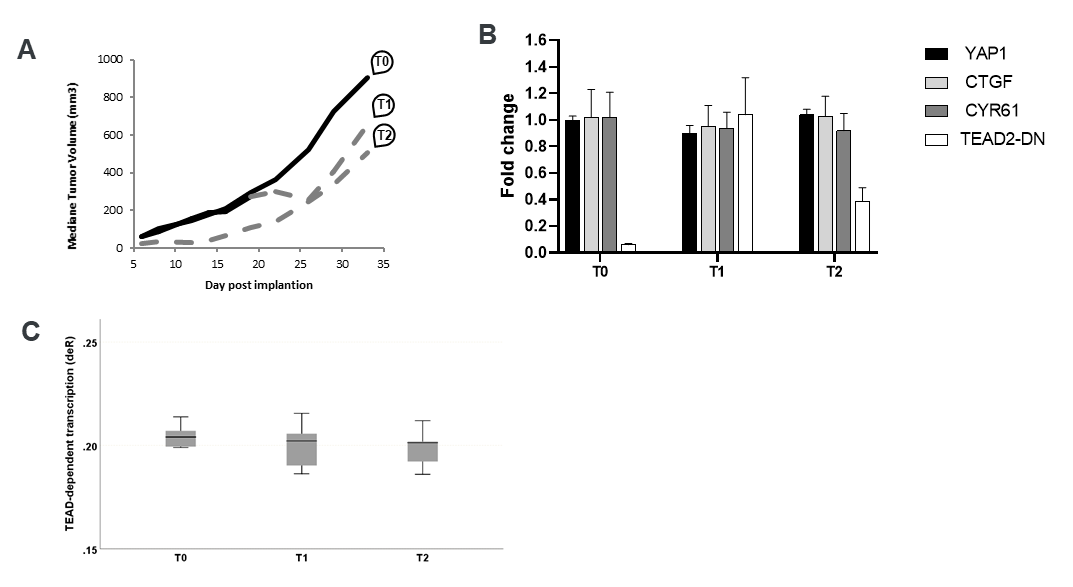


**Figure S3: Pharmacodynamic analysis at the end of the in vivo tumor growth study of MSTO-211H-TEAD2-DN xenografts**

**A** Tumors were sampled on day 34, groups T0: glucose supplementation from day 0-34 (n=5), T1 = doxycycline supplementation from day 0-34 (n=5) and T2: glucose D0-D19 + doxycycline D19-D34 (n=5). **B** mRNA expression by RTQ PCR. **C** TEAD-dependent transcription (deR)

- **Figure S4**


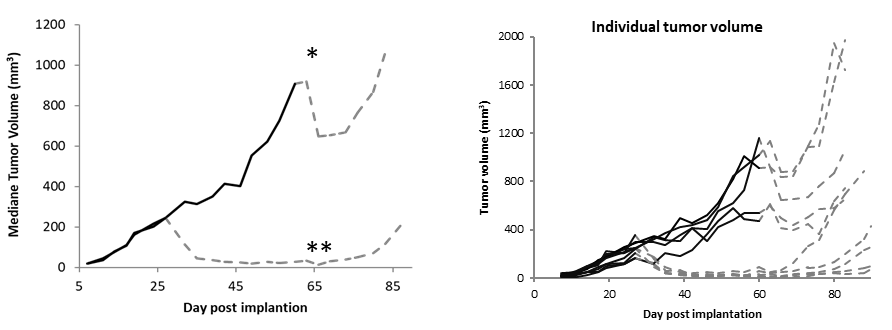


**Figure S4: In vivo tumor growth after long term observation in MSTO-211H-SH-YAP1 xenografts**

**(*)** Doxycycline supplementation on mice bearing established tumors (476 to 1157 mm^3^ at day 60), induced a transitory regression in 5 out of 5 mice, followed by resumption of the tumor growth. **(**)** After tumoral regressions achieved 96% median regression at day 66, tumors began to regrow under SH-YAP pressure.

- **Figure S5**


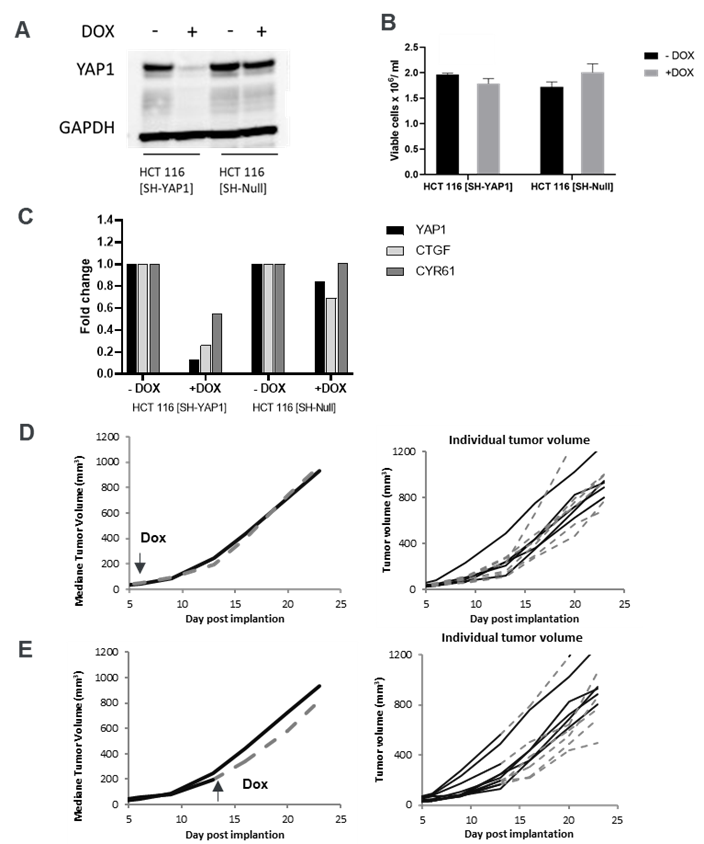


**Figure S5: In vitro and in vivo evaluation of inducible YAP1 knock down effects in Hippo-YAP1 independent HCT116-SH-YAP1 colon cancer cells and xenografts**

**A** Western immunoblot analyses of YAP1 expression in HCT116-SH-YAP cell lysates. GAPDH was used as a loading control. **B** 2D cell viability at 96 h post doxycycline induction. **C** RT-qPCR of YAP1 and the YAP1-TEAD downstream targets CTGF and CYR61 in the HCT116-SH-YAP cell line 72 h post doxycycline induction. **D** In vivo tumor growth in mice supplemented with either doxycycline (black curve) or 5% glucose (dotted grey curve) in drinking water at day 0 post HCT116-SH-YAP1 cell line engraftment. **E** In vivo tumor growth in mice supplemented with 5% glucose (black curve) in drinking water at day 0 post HCT116-SH-YAP1 cell line engraftment and 13 days post HCT116-SH-YAP1 cell line engraftment, half of the mice (n=6) bearing established tumors ranging from 165 to 561 mm^3^ were supplemented with doxycycline in drinking water (dotted grey curve).

- **Figure S6**


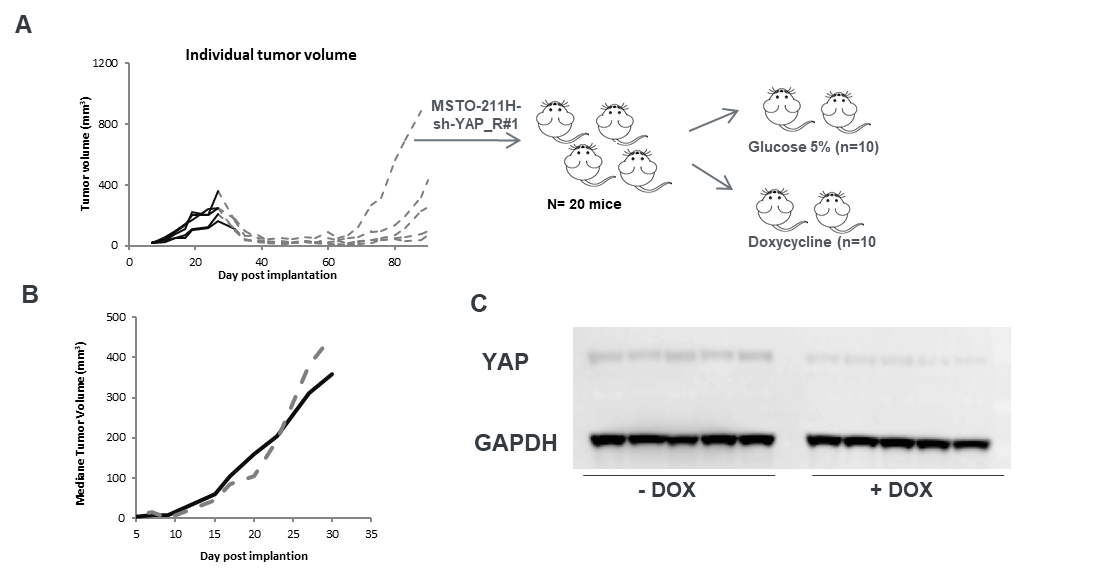


**Figure S6: In vivo tumor growth of the MSTO-211H-SH-YAP1 resistant model**

1. Following long term observations (Figure S3), tumor regrowth was observed under doxycycline treatment, in a tumor from one mouse, named MSTO-211H-SH-YAP1_R#1. Tumor was sampled and grafted into a new set of 20 mice. **B)** MSTO-211H-SH-YAP1_R#1 in vivo tumor growth, mice were supplemented with either 5% glucose (black curve, n=10) or doxycycline (dotted grey curve, n=10) in drinking water at day 0 post engraftment. **C**) YAP1 protein expression in MSTO-211H-SH-YAP1_R#1 xenograft. GAPDH was used as a loading control (n=5 mice/group).

- **Full Western blot images :**
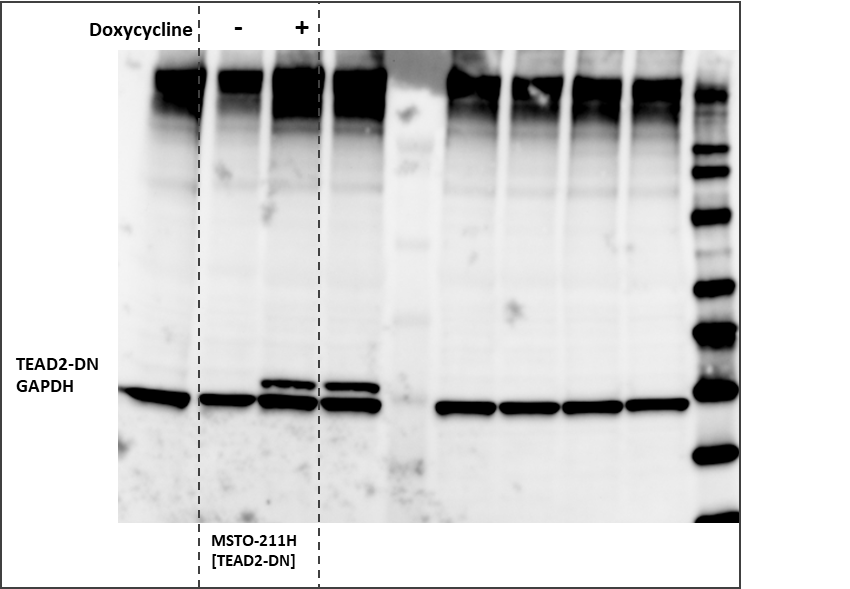


**Figure 1A** : Western immunoblot analyses of TEAD2-DN in MSTO-211H-TEAD2-DN cell line 24 h post doxycycline induction. GAPDH is used as a loading control


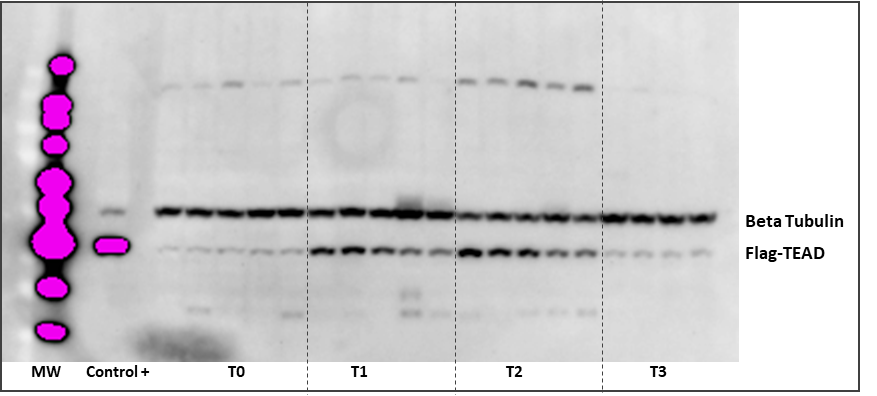


**Figure 3B:** Western blot analysis of TEAD2-DN protein expression. Beta tubulin is used as a loading control

**
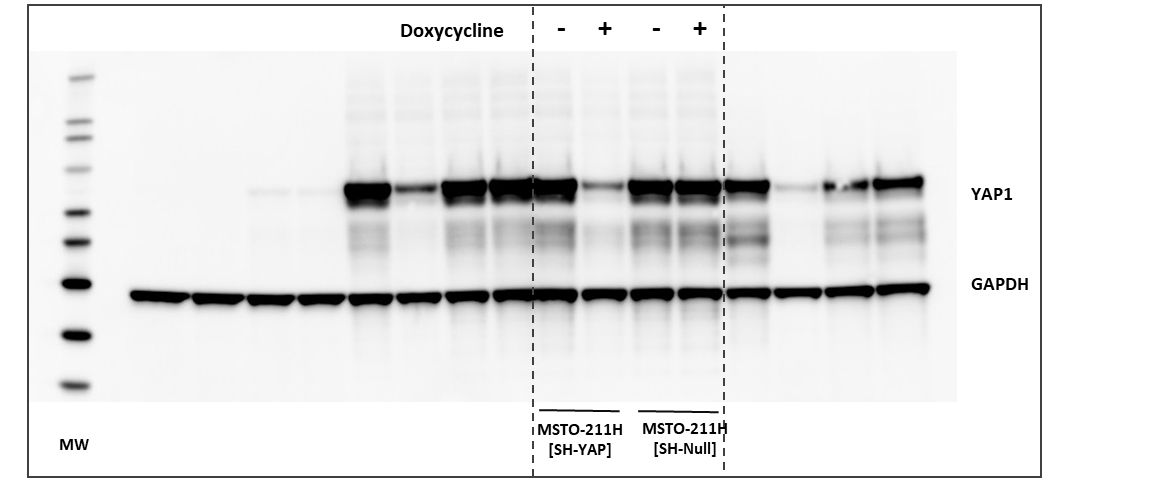
**

**Figure 4B:** Western immunoblot analyses of YAP1 in the MSTO-211H-SH-YAP cell line. GAPDH was used as a loading control.


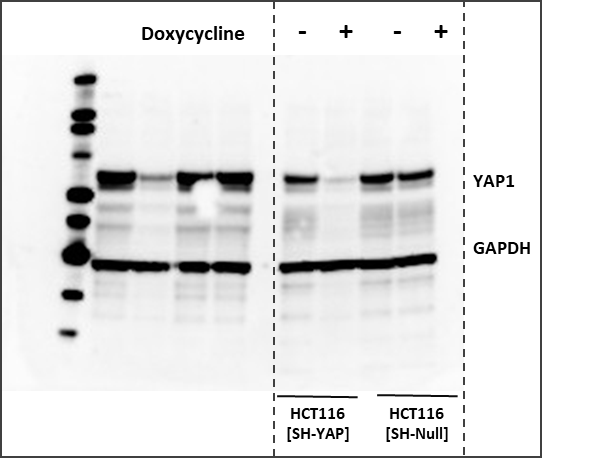


**Figure 5SA :** Western immunoblot analyses of YAP1 expression in HCT116-SH-YAP cell lysates. GAPDH was used as a loading control.


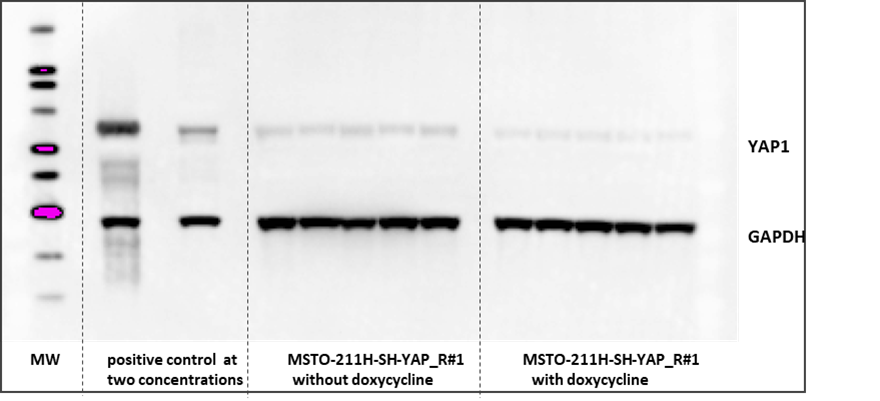


**Figure S6C** Western immunoblot analyses of YAP1 expression in MSTO-211H-SH-YAP1_R#1 xenograft. GAPDH was used as a loading control (n=5 mice/group).
